# Editorial: Bilateral vestibulopathy

**DOI:** 10.3389/fnint.2024.1387066

**Published:** 2024-03-19

**Authors:** Courtney Stewart, William Michael King, Richard Altschuler, Devin McCaslin

**Affiliations:** ^1^LTC Charles S. Kettles VA Medical Center, Research Service, Ann Arbor, MI, United States; ^2^Department of Otolaryngology/Head-Neck Surgery, University of Michigan, Ann Arbor, MI, United States

**Keywords:** vestibular, vestibular hypofunction, bilateral vestibulopathy, inner ear, noise

This Research Topic gathers contributions that highlight characteristics of bilateral vestibulopathy (BVP), tools for studying and tracking this condition, and describe other factors that may impact mobility and cognitive function. We also include studies that highlight the importance of considering environmental factors (e.g., noise exposure history) in subjects that have no known cause of BVP or vestibular hypofunction (VH). The purpose of this topic is to highlight recent advances and propose a potential consideration for idiopathic cases of BVP.

Bilateral vestibulopathy (BVP) was first described in James (1882) and understanding of the symptoms and diagnostic findings of this condition has developed significantly since this time. Diagnostic criteria have emerged and include imbalance and unsteady gait that worsens in darkness or on uneven surfaces, oscillopsia, and bilateral loss of vestibular end-organ and/or peripheral vestibular nerve function.

BVP is not a rare condition. It has been estimated that severe-to-profound BVP occurs in approximately 28:100,00 or 64,000 individuals in the US (Ward et al., 2013). In cases that develop slowly, symptoms may not be perceived due to their slow onset. However, when the symptoms reach a level of severity, increased visual impairment related to oscillopsia, difficulty balancing, loss of self-perception of motion, and fall risk become serious concerns. Generally, at this time, patients are evaluated and diagnosed. Ward et al. (2013) queried patients with BVP about their degree of disability. In that study, 44% of patients stopped driving because of their symptoms and 75% became unemployed. In another study, 90% of respondents reported an impairment in health health-related quality of life (Guinand et al., 2012). The etiology is unclear for almost half of patients evaluated, but in remaining cases causes include surgery, genetic mutations, drug exposure (aminoglycosides and platins), bilateral Meniere's Syndrome, bilateral vascular occlusion, infection, and tumors of the temporal bone. Research is emerging that suggests cumulative noise exposure over one's lifespan may also be a factor underlying this debilitating condition.

Beyond the disabilities noted above, previous work has documented hippocampal atrophy and impaired spatial memory in BVP patients (e.g., Brandt et al., 2005). An elegant study by Maguire et al. (1997) used positron emission tomography (PET) to investigate the hippocampus in London taxi drivers. Gray matter volume (voxel-based morphometry) in the posterior hippocampus of London cab drivers was greater than age-matched controls and the size of this increase was associated with years spent driving a taxi. In this topic, Lee et al. compare canal function and questionnaire data with changes in neural connections and volume of key brain regions. Their observations may be linked to cognitive dysfunction. Results of this work support the importance of routine clinical measures such as questionnaires and horizontal canal testing for diagnosis of BVP, but also highlight the importance of neuroimaging in identifying and monitoring factors that can significantly impact daily functioning, not only due to dizziness and imbalance, but also due to depression and cognitive impairment. This work also demonstrates that in addition to hippocampal atrophy, changes in functional connections that support the salience network are altered (Lee et al.).

Development of questionnaires that are simple to complete and can be interpreted to assess severity and changes related to treatment or progression of BVP are of significant value. Using questionnaires such as the Bilateral Vestibulopathy Questionnaire, described in this topic, are of significant value as a quick high-level assessment tool (van Stiphout et al.).

Hearing status of BVP patients is also investigated in this topic. Moyaert et al. demonstrate that only 21% of patients with BVP have normal hearing and nearly half have profound hearing loss in at least one ear. Within this group, 40% of patients with idiopathic BVL have profound hearing loss and only 17% have normal hearing. An inability to identify a single event that may have caused BVL suggests that in some subjects, BVL develops through cumulative insults over time (e.g., environmental noise exposure).

Canal functional tests are a critical component of BVP assessment. Otolith organ tests also have the potential to provide early diagnosis of idiopathic cases of BV. This may be particularly relevant where cumulative exposure to environmental factors that damage the vestibular periphery are involved. For instance, if noise is shown to be a contributing factor, then the otolith organs are most affected by environmental noise, followed by the semicircular canal cristae (Stewart et al., 2016). In this topic, firefighters, who are exposed to a range of damaging noise frequencies and vibrations were studied, and it was observed that in addition to elevated high-frequency hearing thresholds, firefighters also had reduced cervical vestibular evoked myogenic potential (VEMP) amplitudes and elevated thresholds, versus controls (Snapp et al.). Another contribution to this topic demonstrates the successful generation of reliable and reproducible VEMPs in rats. While this has been done in other studies (e.g., Hsu et al., 2008), development of this method for use in animal models has been difficult. Interestingly, Raciti et al. have reported that ACS-evoked VEMPs in rats are best at 6-10 kHz. Variability in best frequency could be species-dependent; however, this novel finding may change how we view the impact of a larger range of frequencies on vestibular function and dysfunction.

Lastly, this topic characterizes factors that impact walking. It is shown that rats exposed to noise that has been previously shown to causes vestibular injury resulting in physiological deficits (Stewart et al., 2020) walk more slowly in a simple balance beam crossing task (Bartikofsky et al.). It is also demonstrated that even in young, healthy adults, distractions lead to perturbations and errors in gait (Bazzi and Cacace). In subjects with BVP, it is likely that a significantly greater amount of attention must be paid to walking, particularly since reduced cognitive function is observed. Distractions, even very small ones, may lead to falls and serious injuries that impact health, safety, and independence.

We hope that readers will find benefit in this topic's integration of recent studies of BVP, otolith organ dysfunction, and factors that influence mobility.

## Author contributions

CS: Writing – original draft, Writing – review & editing. WK: Writing – original draft, Writing – review & editing. RA: Writing – original draft, Writing – review & editing. DM: Writing – original draft, Writing – review & editing.
